# Assessment of Biophysical Parameters and Acute Lesion Effectiveness in Atrial Fibrillation Ablation Using a Very High-Power Short-Duration Strategy

**DOI:** 10.7759/cureus.109050

**Published:** 2026-05-17

**Authors:** Sana Tahir, Joseph Rogers, Viraj Shetty, Devender Akula

**Affiliations:** 1 Internal Medicine, AtlantiCare Regional Medical Center, Pomona, USA; 2 Electrophysiology, Biosense Webster, Pomona, USA; 3 Medicine, Victoria Hospital, Bengaluru, IND; 4 Electrophysiology, AtlantiCare Regional Medical Center, Pomona, USA

**Keywords:** atrial fibrillation ablation, atrial fibrillation (af), catheter stability, contact force, high-power short-duration, impedance drop, maximum temperature

## Abstract

Background: Radiofrequency ablation (RFA) therapy is frequently performed to maintain sinus rhythm in patients with atrial fibrillation (AF).

Objective: The purpose of this study was to assess the association between biophysical parameters (average contact force, maximum temperature, catheter stability, and impedance drop) and acute lesion effectiveness using a very high-power short-duration (90 W, 4 seconds, QDOT catheter, Biosense Webster, Irvine, California) ablation strategy for atrial fibrillation.

Methods: We analyzed 986 ablation lesions from 13 consecutive patients who underwent RFA between June and September 2024. After the pulmonary vein antral isolation (PVAI) lesion set, each lesion was assessed for the presence or absence of capture with high-output pacing (10 mA, 2 ms). Group A included lesions with no local capture, and Group B included lesions with local capture. Lesion characteristics were obtained from data stored in the CARTONET cloud-based platform (Biosense Webster). For each lesion, average contact force, maximum temperature, impedance drop, and catheter stability were analyzed. Statistical analysis was performed using independent t-tests, treating lesions as the unit of analysis.

Results: Group A had more ablation lesions, higher contact force, a higher maximum temperature, and better stability compared with Group B (Group A vs Group B: 818 vs 168 lesions, p < 0.01; 19.47 g vs 17.58 g, p < 0.01; 44.96 °C vs 40.64 °C, p < 0.01; 1.59 mm vs 9.58 mm, p < 0.01). Group B had a higher impedance drop than Group A (10.35 Ω vs 8.27 Ω, p < 0.01). There were no major complications in either group at the 30-day follow-up.

Conclusion: Higher average contact force, higher temperature, and better stability were associated with successful lesion delivery utilizing a very high-power short-duration ablation strategy. In this cohort, an impedance drop beyond 8 Ω did not appear to be significantly associated with acute electrical isolation.

## Introduction

Atrial fibrillation (AF) is the most common arrhythmia, and its burden has been increasing globally due to the aging population, lifestyle choices such as physical inactivity, alcohol use, illicit drug use, and smoking, as well as medical conditions such as chronic obstructive pulmonary disease, chronic kidney disease, valvular heart disease, hypertension, sick sinus syndrome, hyperthyroidism, and obstructive sleep apnea [1]. AF prevalence has increased threefold over the last 50 years, as shown by the Framingham Heart Study [2]. AF is an independent risk factor for stroke, heart failure, and mortality [3]. AF treatment options include rate control and rhythm control strategies using anti-arrhythmic drugs (AADs) or catheter ablation (CA).

A seminal study by Haïssaguerre et al. provided evidence that AF is triggered by pulmonary vein ectopy, leading to the development of widely practiced pulmonary vein antral isolation (PVAI), which now forms the core of the majority of AF ablation procedures [4]. AF ablation is successful when there is creation of a contiguous, transmural, and electrically isolated ablation scar around the pulmonary veins without collateral tissue damage [5]. Previously, low-power, long-duration (LPLD) (25-50 W, 20-40 seconds) radiofrequency ablation (RFA) was employed until 2020, when high-power, short-duration (HPSD) (90 W, 4 seconds) RFA was performed [6]. The Q-Efficiency Trial revealed that the new QDOT Micro catheter (Biosense Webster, Johnson & Johnson, Irvine, California) in HPSD mode alone or in combination with LPLD mode is highly efficient and effective without compromising safety [7].

Prior studies have shown that CA is more effective than AAD treatment in AF patients [8,9]. Despite CA being considered the preferred therapy, multiple studies have shown that patients still have recurrence. The Catheter Ablation vs Anti-Arrhythmic Drug Therapy for Atrial Fibrillation (CABANA) trial showed that only 50% of AF patients who underwent ablation were AF-free at three years after the procedure, with up to 17% requiring repeat ablation procedures [10]. Given the high recurrence rate, lesion durability is crucial to the long-term success of CA. Prior studies have identified several RFA lesion parameters that predict effective ablation. Our study aims to assess ablation characteristics, including contact force, maximum temperature, catheter stability, and impedance drop, during HPSD ablation.

## Materials and methods

After Institutional Review Board (IRB) approval, the study was conducted at AtlantiCare Regional Medical Center in Pomona, New Jersey, between June and September 2024. This was a prospective observational study, and the HPSD ablation strategy was used as part of routine clinical practice (Table 1).

**Table 1:** Comparison of low-power long-duration ablation with the very high-power short-duration strategy used in this study. The HPSD approach delivers 90 W of radiofrequency energy for 4 seconds using the QDOT Micro-catheter, compared with conventional low-power long-duration strategies (25-50 W for 20-40 seconds). This technique is designed to produce more focused resistive heating and potentially improve lesion formation while limiting collateral tissue injury. HPSD: high-power short-duration, LDLD: low-power long-duration.

Feature	LPLD Ablation	HPSD Ablation
Power	~25-50 W	~90 W
Duration	20-40 seconds	4 seconds
Lesion type	Longer conductive heating Larger thermal spread	More resistive heating More focused lesion

A systematic assessment of 986 ablation lesions was conducted on 13 consecutive patients who underwent RFA for AF. As shown in Table 2, patient characteristics, including gender, mean age, mean BMI, type of AF (permanent vs paroxysmal), average left atrial (LA) volume, and AF risk factors, were collected. 

**Table 2:** Baseline characteristics of the study population (n=13). Continuous variables are presented as mean ± standard deviation (SD). Categorical variables are presented as n (%). Left ventricular ejection fraction data were available for 12 of the 13 patients. BMI: body-mass index, AF: atrial fibrillation, LA: left atrium, LVEF: left ventricular ejection fraction, AAD: anti-arrhythmic drug, SD: standard deviation.

Variable	Frequency (n)	Percent (%)
Age (years ± SD)	67 ± 6	
BMI (kg/m^2^ ± SD)	26.9 ± 6	
Male	9	69.2
Female	4	30.8
Paroxysmal AF	9	69.2
Permanent AF	4	30.8
Diabetes mellitus	5	38.5
Hypertension	10	76.9
Ischemic cardiomyopathy	1	7.7
Non-ischemic cardiomyopathy	2	15.4
Cerebrovascular accident	2	15.4
Obstructive sleep apnea	2	15.4
Myocardial infarction	0	0
LA volume (mL/mm^2 ^± SD)	38.78 ± 16.83	
LVEF > 50%	9	69.2%
LVEF 40-45%	1	7.7%
LVEF < 30%	2	15.4%
1 Rate control drug	7	53.8%
2 Rate control drugs	1	7.7%
1 AAD	6	46.2%
2 previous AAD	2	15.4%

Patients were selected using consecutive sampling, including all eligible patients undergoing RFA for AF during the study period. Inclusion criteria included adult patients (≥18 years) with paroxysmal or persistent AF undergoing first-time PVAI using an HPSD ablation strategy. Exclusion criteria included patients undergoing redo ablation procedures, those with incomplete procedural data, or those in whom lesion-level CARTONET (Biosense Webster, Irvine, California) data were not available for analysis.

In the study, catheter stability was defined as the maximal deviation (in mm) of the catheter tip from the target point during the four seconds of radiofrequency energy application. Peak temperature is the highest temperature measured by the thermocouple at the catheter tip during energy delivery. The acute effectiveness of the created lesion was determined based on the inability to achieve local capture (Group A) during high-output pacing (10 mA, 2 ms).

**Figure 1:**
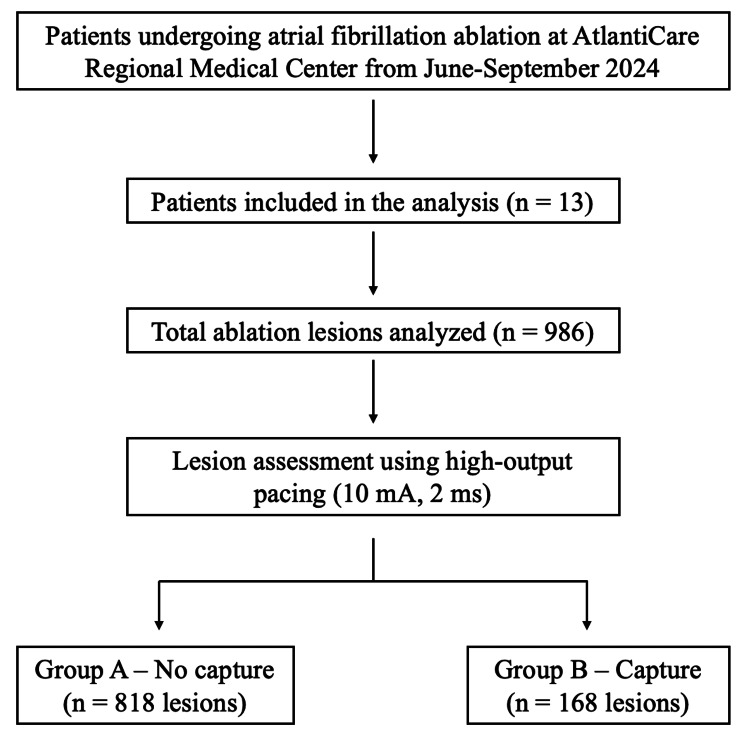
Study flow diagram illustrating patient inclusion and lesion classification. Consecutive patients undergoing first-time radiofrequency catheter ablation for atrial fibrillation between June and September 2024 were included. A total of 13 patients and 986 ablation lesions were analyzed. Lesions were classified according to pacing response using high-output pacing (10 mA, 2 ms) immediately following lesion delivery. Figure created using Microsoft PowerPoint (Microsoft Corporation, Redmond, Washington).

Immediately following PVAI, each lesion was systematically tested for the presence or absence of capture with high-output pacing (10 mA, 2 ms) to evaluate lesion effectiveness. Pacing was performed intraprocedurally, and the response was used to classify lesions into two groups: Group A (absence of local capture) and Group B (presence of local capture), as depicted in Figure 1. Ablation lesion parameters were obtained from the CARTONET cloud-based platform, which provided post-procedural data analysis. The following lesion parameters were studied: average contact force (g), maximum temperature (°C), impedance drop (Ω), and stability (mm).

Statistical analysis was conducted using IBM SPSS Statistics for Windows (IBM Corp., Armonk, New York) software. Data for continuous variables were presented as mean ± standard deviation (SD). The unit of analysis for this study was the individual ablation lesion (n = 986). Comparisons between continuous variables were conducted using the independent-samples t-test. Considering the exploratory approach in this study, lesions were treated as independent observations in determining the biophysical patterns related to acute pacing response. No validated clinical scoring systems or scales were used in this study. 

## Results

A total of 13 patients were included in the study, of whom 69.2% were male and 30.8% were female. Paroxysmal AF was observed in 69.2% of patients, and 30.8% had permanent AF. The average LA volume was 38.78 mL/mm², and the average body mass index (BMI) was 26.9 kg/m². Comorbidities included diabetes mellitus in 38.5%, hypertension in 76.9%, ischemic cardiomyopathy in 7.7%, non-ischemic cardiomyopathy in 15.4%, previous cerebrovascular accident in 15.4%, and obstructive sleep apnea in 15.4% of patients. None of the patients had a history of myocardial infarction (MI). Regarding left ventricular function, among the 12 patients with available echocardiographic data, 69.2% had an ejection fraction (EF) greater than 50%, 7.7% had an EF of 40-45%, and 15.4% had an EF of less than 30%. Six patients had previously tried one AAD, while two had tried two AADs (see Table 2).

**Figure 2:**
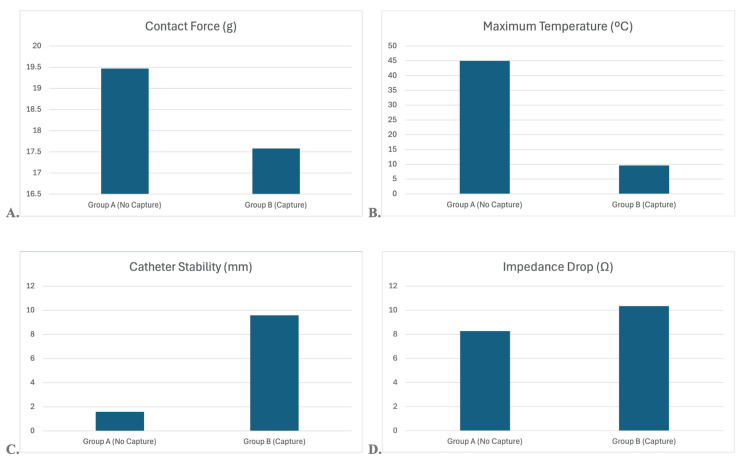
A comparison of ablation parameters between lesion groups. (A) Contact force, (B) maximum temperature, and (C) catheter stability were significantly different between lesions without capture (Group A) and lesions with capture (Group B). (D) Impedance drop beyond 8 Ω appeared not to be predictive. Figure created using Microsoft Excel (Microsoft Corporation, Redmond, Washington).

In this study, lesions in Group A (non-capturing) showed higher values of specific ablation parameters than those in Group B (capturing), suggesting that these parameters correlate with acute electrical isolation (Table 3 and Figure 2). Specifically, a significantly higher number of lesions were observed in Group A than in Group B (818 vs 168 lesions; p < 0.01). Group A lesions were created with higher contact force (19.47 g vs 17.58 g; p < 0.01) and achieved higher peak temperatures during ablation (44.96 °C vs 40.64 °C; p < 0.01). Catheter stability was also significantly greater in Group A (1.59 mm vs 9.58 mm; p < 0.01). Interestingly, Group A demonstrated a smaller impedance drop than Group B (8.27 Ω vs 10.35 Ω; p < 0.01). At the 30-day follow-up, there were no major complications, including pericardial effusion, tamponade, esophageal injury, or pulmonary vein stenosis. 

**Table 3:** A comparison of ablation parameters between Group A and Group B. Comparisons between groups were performed using independent sample t-tests.

Parameter	Group A (No Capture)	Group B (Capture)	p-value
Number of lesions	818	168	< 0.01
Contact force (g)	19.47	17.58	< 0.01
Maximum temperature (ºC)	44.96	40.64	< 0.01
Catheter stability (mm)	1.59	9.58	< 0.01
Impedance drop (Ω)	8.27	10.35	< 0.01

## Discussion

A successful ablation lesion has been associated with several biophysical parameters, such as contact force, contact duration, lesion size, power delivery, impedance drop, catheter stability, peak temperature at lesion formation, and force-time integral. In our study, all 986 ablation lesions were created with a fixed high power of 90 W and a fixed short contact duration of 4 seconds. While those variables were fixed, variables including contact force, catheter stability, temperature, and impedance drop were assessed. Lesions without local capture during high-output pacing (Group A) were associated with higher contact force, greater catheter stability, and higher temperature. Interestingly, in Group B, despite a higher impedance drop, the lesions remained electrically active.

Association of contact force with lesion success

Catheter contact is an important variable for obtaining an effective RFA lesion [11]. Improved contact indicates increased interaction between the electrode surface and the myocardium, leading to a smaller amount of radiofrequency loss in the blood pool [11]. Increased contact force leads to increased lesion size. Ablation lesions have been reported to be more effective when delivered with contact force in the range of 10 to 20 g, whereas lesions with contact force over 40 g increase the risk of tissue injury, char, steam pops, and thrombi [11]. A 10 to 22 g contact force lesion has been shown to acutely block pulmonary vein reconnection with a likelihood greater than 95% [12]. In this study, Groups A and B were both in that range, with Group A having a higher contact force (19.47 g vs 17.58 g), which corresponded to more successful lesions.

Association of catheter stability with lesion success

Catheter stability is also an important parameter in defining a successful ablation lesion. The stability of the catheter (i.e., the extent of catheter movement across the tissue and temporal constancy) also correlates with lesion size. Catheter stability is influenced by respiratory motion. In a multicenter prospective study, catheter stability of >2 mm had an AF recurrence rate of 29%, while for the <2 mm group it was 14% [13]. In our study, Group A had significantly more stable lesions (1.59 mm vs 9.58 mm), which corresponded to more successful ablation lesions. This finding highlights the importance of operator techniques in minimizing catheter mobility, robotic-assisted guidance, and ventilation mode in optimizing lesion formation.

Association of maximum temperature with lesion success

Local maximum temperature is another crucial biophysical parameter that determines a durable lesion. Higher maximum temperatures have been shown to be more effective, with the majority of studies proposing a cutoff point at around 65 to 70 °C, above which the risk of steam pop increases [5,14]. In this study, Group A lesions had a higher maximum temperature than Group B (44.96 °C vs 40.64 °C), indicating more durable lesions.

Association of impedance drop with lesion success

In this HPSD group, a greater reduction in impedance was not necessarily associated with electrical silence, indicating that conventional impedance thresholds based on low-power settings cannot be readily extrapolated to the 90 W/4-second mode. A decrease in impedance of 10.4 Ω has been predicted to result in a 67% likelihood of arrhythmia-free survival [13]. However, in our study, we found that Group B lesions, despite an average impedance drop of 10.35 Ω, remained electrically active. This finding suggests that impedance decrease alone may not be predictive of successful lesions. Given the small sample size and single-center design, this finding needs to be further studied in larger multicenter populations. In conclusion, our findings highlight the need for multifactorial lesion assessment rather than sole reliance on an isolated parameter.

Limitations

Our study has several limitations. It was a single-center, single-operator study with a small sample of 13 patients, which limits the generalizability of the findings. While the analysis of 986 lesions provides a high N value for procedural observation, our statistical approach treated these as independent data points. This means that the clustering effect of multiple lesions within a single patient was not accounted for, making the p-values exploratory. In addition, although there were no significant complications at the 30-day follow-up, long-term clinically meaningful outcomes, such as AF recurrence and lesion durability, are not known. A longer follow-up period and redo procedures are necessary to better understand the long-term effects of these lesion characteristics. The study does not account for patient-specific variations such as atrial wall thickness, tissue composition, and conductivity, or other factors such as catheter orientation that might influence lesion efficacy.

## Conclusions

This study suggests the usefulness of a multiparameter evaluation in assessing acute lesion effectiveness during HPSD RFA. Higher contact force, better catheter stability, and higher peak temperature all demonstrated strong correlations with acute successful lesion formation. In this cohort, impedance drop did not strongly correlate with acute lesion success. Given the small sample size and exploratory nature of this analysis, these findings serve as a basis for larger prospective trials to further validate the clinical impact of these biophysical trends. The study indicates that traditional parameters associated with lesion durability with LPLD may similarly relate to lesion durability with HPSD. Longer follow-up with a larger patient population is necessary to confirm these findings, optimize ablation techniques, and improve arrhythmia-free survival.
